# Composition of Glycosaminoglycans in Elasmobranchs including Several Deep-Sea Sharks: Identification of Chondroitin/Dermatan Sulfate from the Dried Fins of *Isurus oxyrinchus* and *Prionace glauca*

**DOI:** 10.1371/journal.pone.0120860

**Published:** 2015-03-24

**Authors:** Kyohei Higashi, Yoshiki Takeuchi, Ann Mukuno, Hideyuki Tomitori, Masaki Miya, Robert J. Linhardt, Toshihiko Toida

**Affiliations:** 1 Graduate School of Pharmaceutical Sciences, Chiba University, 1-8-1 Inohana, Chuo-ku, Chiba 260-8675, Japan; 2 Faculty of Pharmacy, Chiba Institute of Science, 15-8 Shiomi-cho, Choshi, Chiba 288-0025, Japan; 3 Natural History Museum and Institute, 955-2 Aoba-cho, Chuo-ku, Chiba 260-8682, Japan; 4 Department of Biology, Center for Biotechnology and Interdisciplinary Studies, Rensselaer Polytechnic Institute, 110 8th Street, Troy, New York, United States of America; University of Patras, GREECE

## Abstract

Shark fin, used as a food, is a rich source of glycosaminoglyans (GAGs), acidic polysaccharides having important biological activities, suggesting their nutraceutical and pharmaceutical application. A comprehensive survey of GAGs derived from the fin was performed on 11 elasmobranchs, including several deep sea sharks. Chondroitin sulfate (CS) and hyaluronic acid (HA) were found in *Isurus oxyrinchus*, *Prionace glauca*, *Scyliorhinus torazame*, *Deania calcea*, *Chlamydoselachus anguineus*, *Mitsukurina owatoni*, *Mustelus griseus* and *Dasyatis akajei*, respectively. CS was only found from *Chimaera phantasma*, *Dalatias licha*, and *Odontaspis ferox*, respectively. Characteristic disaccharide units of most of the CS were comprised of C- and D-type units. Interestingly, substantial amount of CS/dermatan sulfate (DS) was found in the dried fin (without skin and cartilage) of *Isurus oxyrinchus* and *Prionace glauca*. ^1^H-NMR analysis showed that the composition of glucuronic acid (GlcA) and iduronic acid (IdoA) in shark CS/DS was 41.2% and 58.8% (*Isurus oxyrinchus*), 36.1% and 63.9% (*Prionace glauca*), respectively. Furthermore, a substantial proportion of this CS/DS consisted of E-, B- and D-type units. Shark CS/DS stimulated neurite outgrowth of hippocampal neurons at a similar level as DS derived from invertebrate species. Midkine and pleiotrophin interact strongly with CS/DS from *Isurus oxyrinchus* and *Prionace glauca*, affording *K_d_* values of 1.07 nM, 6.25 nM and 1.70 nM, 1.88 nM, respectively. These results strongly suggest that the IdoA-rich domain of CS/DS is required for neurite outgrowth activity. A detailed examination of oligosaccharide residues, produced by chondroitinase ACII digestion, suggested that the IdoA and B-type units as well as A- and C-type units were found in clusters in shark CS/DS. In addition, it was discovered that the contents of B-type units in these IdoA-rich domain increased in a length dependent manner, while C- and D-type units were located particularly in the immediate vicinity of the IdoA-rich domain.

## Introduction

Glycosaminoglycans (GAGs) are a group of structurally related polysaccharides, found as the carbohydrate moieties of proteoglycans (PGs) or as free chains in the case of hyaluronic acid (HA). The GAG components of PGs are linear, sulfated polysaccharides containing hexosamine and uronic acid (or galactose (Gal)) disaccharide repeating sequence and include chondroitin sulfate (CS), dermatan sulfate (DS), heparin (HP), heparan sulfate (HS), keratan sulfate (KS) [1]. CS is composed of repeating disaccharide unit, [-4) GlcA (β1–3) GalNAc (β1-]_n_, where GlcA is glucuronic acid and GalNAc is *N*-acetylgalactosamine. DS is biosynthesized through the action of glucuronyl C5-epimerase on CS, converting its GlcA to the CS epimer, iduronic acid (IdoA). GAGs (CS, DS, HP, and HS) are covalently attached to serine residues of a core protein through a linkage region tetrasaccharide GlcA-Gal-Gal-Xyl (where Xyl is xylose). The repeating disaccharides of CS and DS can be sulfated at the C-2 position of GlcA or IdoA and at the C-4 and/or C-6 positions of GalNAc in animals [2–6]. The content of IdoA in a DS chain can range from a single IdoA residue to nearly 100% IdoA residues. The disaccharide units in CS and DS are classified into five groups, GlcA/IdoA-GalNAc (4S) (A-type unit), GlcA/IdoA-GalNAc (6S) (C-type unit), GlcA/IdoA (2S)-GalNAc (4S) (B-type unit), GlcA/IdoA (2S)-GalNAc(6S) (D-type unit) and GlcA/IdoA-GalNAc (4S, 6S) (E-type unit) and the physiological function of CS and DS, such as differentiation, migration, tissue morphogenesis, immune response, blood clotting and wound repair, depend on the binding between their sulfated oligosaccharides and the functional proteins in mammals [5,7–9].

GAGs have been purified from various animal tissues and their structures characterized to understand their biological distribution and to explore new sources of polysaccharides containing unique structures. GAGs are widely distributed throughout the animal kingdom, and invertebrate species are known to represent rich sources of these sulfated polysaccharides [10]. The composition of IdoA in DS varies in animal species, tissues, and cell types. For example, CS/DS derived from porcine skin, eel skin [11], marine clam [12], ascidian [13,14], hagfish notochord [15], sea urchin [16] are comprised of repeating disaccharide units having a high IdoA content (more than 80%) and the DS possess both anticoagulant and neurite outgrowth activities [17]. In contrast, CS/DS isolated from horse aorta [18], embryonic pig brain [19], mouse brain [20], mouse skin [21] and HEK293 cells [22] have a low content of IdoA, and can associate with growth factors such as pleiotrophin and midkine. These differences in the IdoA contents of CS/DS chains are attributable to the levels of DS epimerases (DS-epi1 and -epi2) as well as DS-specific 4-*O*-sulfotransferase (D4ST1) in Golgi apparatus of the cells comprising in each animal species and tissue [2], however, the determinant responsible for the control of epimerization activity in CS/DS biosynthesis is still unknown.

Recently, the structural sequence of bikunin CS and porcine skin decorin DS have been solved using FT-ICR-MS analysis and the positions of the sulfate groups in bikunin CS and of the IdoA residues in decorin DS localized to particular domains within the GAG chain [23,24]. Bikunin CS and decorin DS chains are composed of almost entirely of A-type units. From these studies it still remains unclear whether chains of CS/DS that contain mixtures of A-, B-, C-, D- and E-type units can be effectively sequenced.

In the current study, a composition analysis of GAGs from the fin of 11 kinds of elasmobranchs, including deep-sea sharks, was systematically performed. As a result, we have identified CS/DS containing substantial quantities of A-, C-type units and small amount of B-, D-, E-units from dried shark fin in *Isurus oxyrinchus* and *Prionace glauca*, and have analyzed both sequence and neurite outgrowth activity.

## Materials and Methods

### Ethics statement

H. Tejima, a fisherman, belongs to the Amaha Fisheries Cooperative in Futtsu, Chiba, Japan. Fins from deep-sea elasmobranchs were kindly provided by him, before being processed as food materials. No specific permissions were required to collect elasmobranchs for the study area and the collected elasmobranchs did not involve any endangered or protected species.

All animal experiments were approved by the Institutional Animal Care and Use Committee of Chiba University and carried out according to the guidelines for Animal Research of Chiba University.

### Materials

Dried fins (without skin and cartilage) of *Isurus oxyrinchus* and *Prionace glauca* and raw fin (without skin) of *Prionace glauca* were kindly provided by Mrs. T. Mano and T. Wada (Nihon Pharmaceutical Co. Ltd.). Deep-sea elasmobranchs (sharks and rays) were collected by H. Tejima through gill net fisheries at the mouth of Tokyo Bay off Kanaya, Chiba, Japan (35.17°N, 139.79°E; 200–300 m depths). Fins from those elasmobranchs were kindly provided by H. Tejima, before being processed as food materials. Actinase E was from **Kaken** Pharmaceutical Co., Ltd., Tokyo, Japan. Chondroitinase ABC (ChaseABC) from *Proteus vulgaris*, chondroitinase ACII (ChaseACII) from *Arthrobacter aurescens*, unsaturated disaccharides (ΔDi-0S, ΔDi-4S, ΔDi-6S, ΔDiUA-2S, ΔDi-diS_E_, ΔDi-diS_B_, ΔDi-diS_D_, ΔDi-TriS) and CS-E were purchased from Seikagaku Corp., Tokyo, Japan. Dialysis membrane for desalting was from Spectrum Medical (Los Angelis, CA, USA).

### Isolation of glycosaminoglycans from shark fins

**Fig 1:**
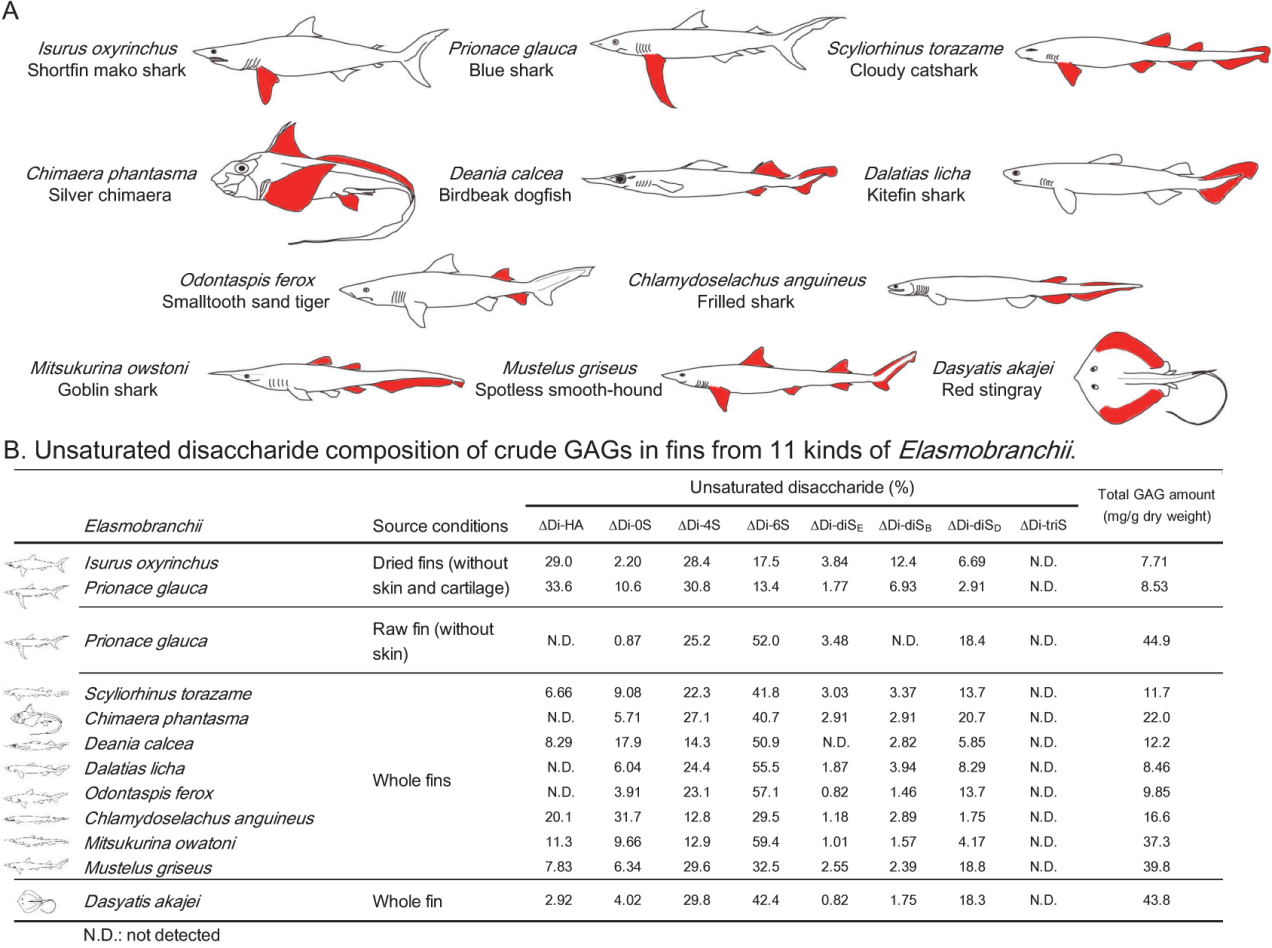
GAG components from various kinds of *Elasmobranchii*. (A) Depiction of 11 kinds of *Elasmobranchii*. Position of fin samples used in this study are shown in red. (B) Determination of disaccharide compositions of GAGs. Purification and unsaturated disaccharide analysis were performed as described under “Materials and Methods”. Unsaturated disaccharide analysis was repeated twice with reproducible results.

Shark fins (Fig. 1A) were lyophilized to dryness. Dried samples (∼3–10 g) were cut into small pieces that were homogenized with 4-volumes of acetone overnight. The precipitates obtained by centrifugation were proteolyzed at 45°C with actinase E (10 mg/g dry powder) in 50 mM Tris acetate (pH 8.0) for 18 h. After the proteolysis, β-elimination was performed with 0.5 M NaOH containing 0.3 M sodium borohydrate (20 mL/g dry sample) at 4°C for 18 h. Samples were treated with 5% perchloric acid and centrifuged, supernatant was dialyzed against water at 4°C and crude GAGs were precipitated by addition of cetylpyridinium chloride (CPC, final concentration, 0.1%) containing 0.03 M NaCl for 3 h at 4°C. The GAG-CPC complex was collected by centrifugation at 2,300 × g for 15 min. The precipitate was washed twice with 0.1% CPC. Crude GAGs were extracted from the GAG-CPC complex by addition of 2.5 M NaCl, and the mixture was centrifuged at 2,300 × g for 15 min. Crude GAGs were precipitated from the supernatant by addition of 4- volumes of cold ethanol for 16 h at 4°C and were collected by centrifugation at 2,300 × g for 15 min. Finally, the collected crude GAGs were dissolved in water, dialyzed against water, and freeze-dried. The GAG recovery for each sample is shown in Fig. 1B.

Crude GAG (∼10–20 mg of dry powder) in 2 mL water was applied at a flow rate 5 mL/min on a HiPrep DEAE FF (16 mm i.d. × 100 mm, obtained from **GE Healthcare Europe GmbH**) and fractionated to prepare highly sulfated CS/DS oligosaccharides. The eluents were (A) 50 mM sodium phosphate, (B) 2.0 M NaCl in 50 mM sodium phosphate, respectively. The gradient program was 0–20 min (5% B), 20–120 min (5–100% B), and 120–140 min (100% B). Fractionated samples were collected at 20 min-intervals, dialyzed, freeze-dried and kept stored at 4°C.

### High performance liquid chromatography

Disaccharide composition analysis of GAGs was performed as follows. Crude GAGs (5 μg) or fractionated CS (0.5 μg) were incubated in reaction mixture (35 μL) which was contained 28.6 mM Tris acetate (pH 8.0), 50 mIU of ChaseABC, 50 mIU of ChaseACII, respectively. After 16 h at 37°C, depolymerized samples were boiled and evaporated, re-suspended in 10 μl of water. Unsaturated disaccharides afforded on digestion with chondroitinase were determined by a reversed phase ion-pair chromatography with sensitive and specific post-column detection with minor modifications [25]. A gradient was applied at a flow rate of 1.0 ml/min on Senshu Pak Docosil (4.6 × 150 mm; Senshu Scientific, Tokyo, Japan) at 60°C. The eluent buffers were as follows: A, 10 mM tetra-*n*-butylammonium hydrogen sulfate in 12% methanol; B, 0.2 M NaCl in buffer A. The gradient program was as follows: 0–10 min (1% B), 10–11 min (1–10% B), 11–30 min (10% B), 30–35 min (10–60% B), and 35–40 min (60% B). Aqueous (0.5% (w/v)) 2-cyanoacetamide solution and 1 M NaOH were added to the eluent at the same flow rates (0.5 ml/min) by using a double plunger pump. The effluent was monitored fluorometrically (excitation, 346 nm; emission, 410 nm). Measurement of HA contents in crude GAGs was performed according to the method of Toyoda *et al*. [26]. High-performance size exclusion liquid chromatography (HPSEC) was performed as described previously [27].

### Effect of chondroitin/dermatan sulfate from shark fin on neurite outgrowth

GAG pre-coating in 8-well chamber slide and evaluation of CS on neurite outgrowth were performed according to the method of Mikami *et al*. [28] and Fongmoon *et al*. [29] with minor modifications. Briefly, 8-well chamber slides (Nunc) were pre-coated with 50 μg/ml of poly-DL-ornithine in 0.1 M sodium borate (pH 8.0) and then coated CS at the specified concentration at 4°C overnight. The mice were anesthetized with isoflurane, and the embryos were dissected out. The hippocampus of embryonic day 16 (E16) mice were dissected and treated with minimum essential medium alpha (MEMα) containing 0.5% glucose, 0.01% DNase I and 0.25% trypsin. After incubation for 10 min at 37°C, cells were mildly dissociated and fetal bovine serum was added to a final concentration of 20%. The cells, recovered by centrifugation, were re-suspended with neurobasal medium containing B27 supplement, 2 mM glutamine, 25 μM glutamate and plated 16,000 cells/cm^2^ on each well pre-coated with a defined substrate, and cultured for 18 h. Almost of the neurons cultured for 18 h on the P-ORN-coated control coverslip had some short neurite (<20 μm). The cultured cells were fixed with 4% paraformaldehyde for 30 min, washed three-times with phosphate buffered saline and permeabilized with 0.2% Triton X-100 for 30 min at the room temperature. The neurites were immunostained with anti-microtuble-associated protein 2 (Sigma) and anti-neurofilament antibodies (Sigma), and then the antibodies were detected using a Vectastain ABC kit (Vector Laboratories Inc., Burlingame, CA) with 3, 3’-diaminobenzidine as a chromogen. The stained cells on each well observed under an Olympus BX51 microscope and images were acquired directly with a cooled CCD camera DP70 (Olympus). Fifty cells with at least one neurite longer than the cell body were chosen at random to determine the length of the longest neurite. At least six independent experiments per condition were carried out.

### Surface plasmon resonance (SPR)

Recombinant growth factors such as human pleiotrophin (PTN) and midkine (MK) were purchased from Peprotech and R&D systems, respectively. The binding of GAGs to growth factors was measured using a BIA 2000 optical Biosensor (GE Healthcare) as described previously [30]. Briefly, recombinant growth factors were immobilized on a CM5 sensor chip (GE Healthcare) according to the manufacturer’s instructions. GAGs prepared specified concentration was applied to flow cells, and data was analyzed by BIA evaluation software (Version 3.0) using 1:1 Langmuir binding model.

## Results

### Comprehensive analysis of GAGs derived from elasmobranchs

We explored the GAG components in a variety of elasmobranchs including deep-sea sharks and red stingray. Fins of elasmobranchs were collected from shortfin mako shark (*Isurus oxyrinchus*), blue shark (*Prionace glauca*), cloudy catshark (*Scyliorhinus torazame*), silver chimaera (*Chimaera phantasma*), birdbeak dogfish (*Deania calcea*), kitefin shark (*Dalatias licha*), smalltooth sand tiger (*Odontaspis ferox*), frilled shark (*Chlamydoselachus anguineus*), goblin shark (*Mitsukurina owatoni*), spotless smooth-hound (*Mustelus griseus*) and red stingray (*Dasyatis akajei*) (depicted in Fig. 1A). Birdbeak dogfish, kitefin shark, smalltooth sand tiger, frilled shark, goblin shark and spotless smooth-hound are found at depths below 200 m. Crude GAGs were extracted from dried fins (without skin and cartilage) of shortfin mako shark and blue shark, or from whole fins from cloudy catshark, silver chimaera, birdbeak dogfish, kitefin shark, smalltooth sand tiger, frilled shark, goblin shark, spotless smooth-hound and red stingray by actinase E digestion, and recovered by ethanol precipitation. The dried pellets (crude GAGs) of each sharks and red stingray were weighed after dialysis and freeze-drying (Fig. 1B). As a result, 7.7∼44.9 mg GAG/g of dry tissue were recovered. Crude GAGs were analyzed by cellulose acetate membrane electrophoresis and visualized with alcian blue in the presence or absence of chondroitinase ABC (ChaseABC) or chondroitinase ACII (ChaseACII) (S1 Fig.). GAGs including one or two bands corresponding to the migration positions of HA or CS standards disappeared on ChaseABC or ChaseACII digestion. These results indicate that most of the GAG present were CS and HA.

**Fig 2:**
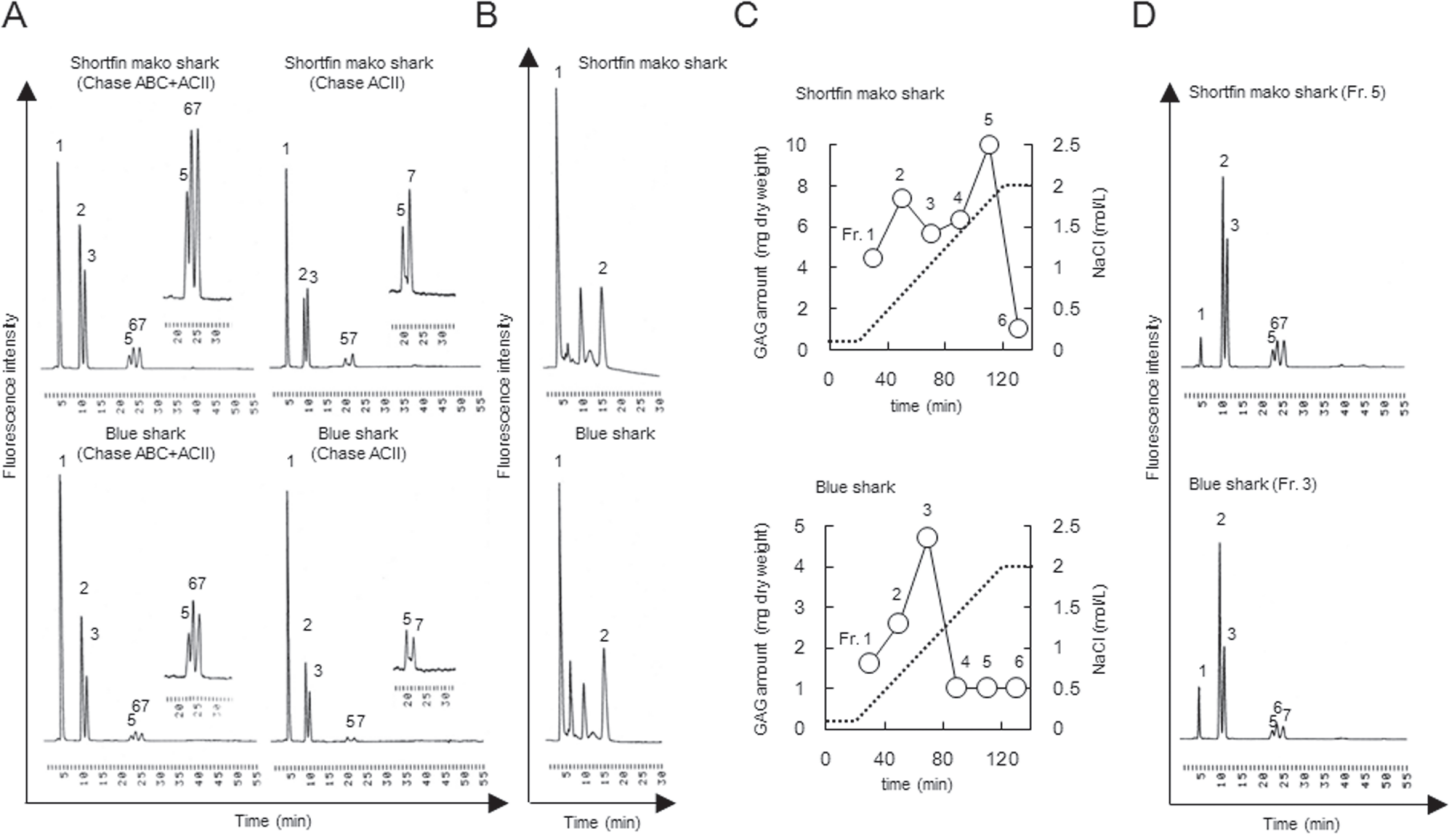
Purification of CS/DS from shortfin mako shark and blue shark fin without skin and cartilage. (A) Determination of CS/DS in shark fins. Briefly, 5 μg of crude GAGs derived from samples were digested by 50 mU of ChaseACII and 50 mU of ChaseABC, and then resulting unsaturated disaccharides were subjected to reversed-phase ion-pair chromatography with sensitive and specific post-column detection. Peaks: 1, ΔDi-0S; 2, ΔDi-4S; 3, ΔDi-6S; 4, ΔDiUA-2S; 5, ΔDi-diS_E_; 6, ΔDi-diS_B_; 7, ΔDi-diS_D_. (B) Determination hyaluronic acid contents in crude GAGs. Unsaturated disaccharides obtained by digestion with ChaseABC and ChaseACII were analyzed by a graphitized carbon column and post column fluorometric detection. Peaks: 1, ΔDi-0S; 2, ΔDi-HA. (C) Purification of CS/DS from crude GAGs. The crude GAGs (10∼20 mg of dry powder) were purified on DEAE-cellulose column as described under “Materials and Methods”. The dried powders obtained, after desalting and lyophilizing the 6 fraction samples, were weighed. (D) Chromatograms of unsaturated disaccharides of CS/DS derived from shortfin mako shark (Fr. 5) and blue shark (Fr. 3). Peaks: 1, ΔDi-0S; 2, ΔDi-4S; 3, ΔDi-6S; 4, ΔDiUA-2S; 5, ΔDi-diS_E_; 6, ΔDi-diS_B_; 7, ΔDi-diS_D_. Experiments were repeated twice with reproducible results.

The disaccharide composition of GAGs derived from dried fins (without skin and cartilage) in shortfin mako shark (*Isurus oxyrinchus*) and blue shark (*Prionace glauca*), after digestion with ChaseABC and/or ChaseACII, was determined using reversed phase ion-pair chromatography with sensitive and specific post-column detection (Fig. 2A). Substantial quantities of ΔDi-0S, ΔDi-4S, ΔDi-6S and small amounts of ΔDi-diS_E_, ΔDi-diS_B_, ΔDi-diS_D_ were observed in GAGs isolated from both tissues. Interestingly, the ratios of ΔDi-4S to ΔDi-6S from dried fins (without skin and cartilage) are between 1.62 (short fin mako shark) and 2.30 (blue shark), whereas that from fin (without skin) from blue shark (*Prionace glauca*) is 0.48 (Fig. 1B). When digestion was performed with only ChaseACII, peak intensities of ΔDi-4S and ΔDi-diS_B_ decreased, indicating that GAGs from shortfin mako shark and blue shark was enriched in DS. Since ΔDi-0S (coming from CS/DS) and ΔDi-HA (coming from HA) could not be separated under our reversed phase ion-pair chromatography conditions, a graphitized carbon column was used to separate ΔDi-0S and ΔDi-HA and a ΔDi-HA peak was observed (Fig. 2B). These results demonstrate that crude GAGs consisted of ∼30% of HA and the remaining GAG was CS/DS (Fig. 1B). Crude GAGs were further fractionated by anion-exchange chromatography, and each fraction was collected, desalted, lyophilized and weighed (Fig. 2C). Compositional analysis of unsaturated disaccharides of both Fr. 5 (shortfin mako shark) and Fr. 3 (blue shark) were carried out and results were shown in Fig. 2D. Unsaturated disaccharides of several kinds of GAGs from other sharks were systematically analyzed as mentioned above (Fig. 1B). These results demonstrate a characteristic disaccharide unit in most of these CS samples consisted of C- and D-type unit.

### Effect of DS derived from shortfin mako shark and blue shark on neurite outgrowth

**Fig 3:**
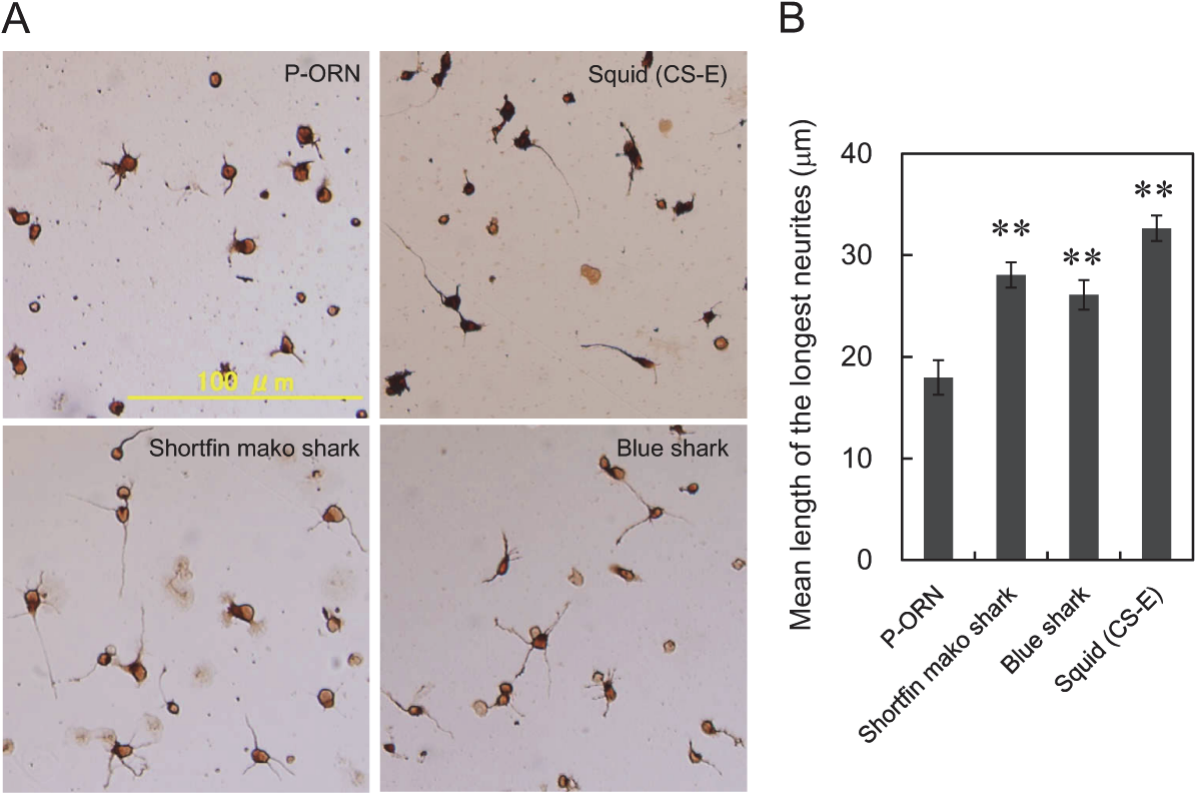
Effect of shark CS/DS from shortfin mako shark (Fr. 5) and blue shark (Fr. 3) on neurite outgrowth. (A) Representative morphological features of E16 hippocampal neurons cultured with shark CS/DS. E16 hippocampal neuronal cells (16000 cells/cm^2^) were cultured for 18h on various substrates coated on P-ORN, fixed and immunostained as described under “Materials and Methods”. (B) The mean length of the longest neurite was measured for more than 50 randomly selected neurons cultured on various substrates (see “Materials and Methods”). The values obtained from six independent experiments are expressed as the mean ±S.E. Mann-Whitney’s U test was used to evaluate the significance of differences between means (**, *p*<0.01).

Highly sulfated chondroitin sulfate like CS-E, CS-D, CS-K and DS from various sources reportedly exhibit neurite outgrowth-promoting (NOP) activity toward E16 embryonic mouse hippocampal neurons [31]. Thus, we examined effect of shark CS/DS from Fr. 5 (shortfin mako shark) and Fr. 3 (blue shark) on neurite outgrowth of E16 mouse hippocampal neurons (Fig. 3). When CS-E was coated (0.5 μg/well) significant stimulation of neurite outgrowth was observed compared with P-ORN-coated control. Hikino *et al*. reported that promotion of neurite outgrowth of E18 rat hippocampal neurons required 2.0 μg/well coated CS-E [17]. However, cell toxicity was observed in the presence of more than 1.0 μg/well of CS-E under our culture conditions. We speculate that some preparations of squid CS-E cause toxicity due to the presence of minor amounts of K-type units [32]. The effective amount of shark CS/DS was next explored and we observed that 1.0 μg/well displayed marked neurite outgrowth-promoting effects, comparable to that observed using 0.5 μg/well of CS-E. However, when 2.0 μg/well of shark CS/DS was coated, significant cell toxicity was observed (data not shown).

**Fig 4:**
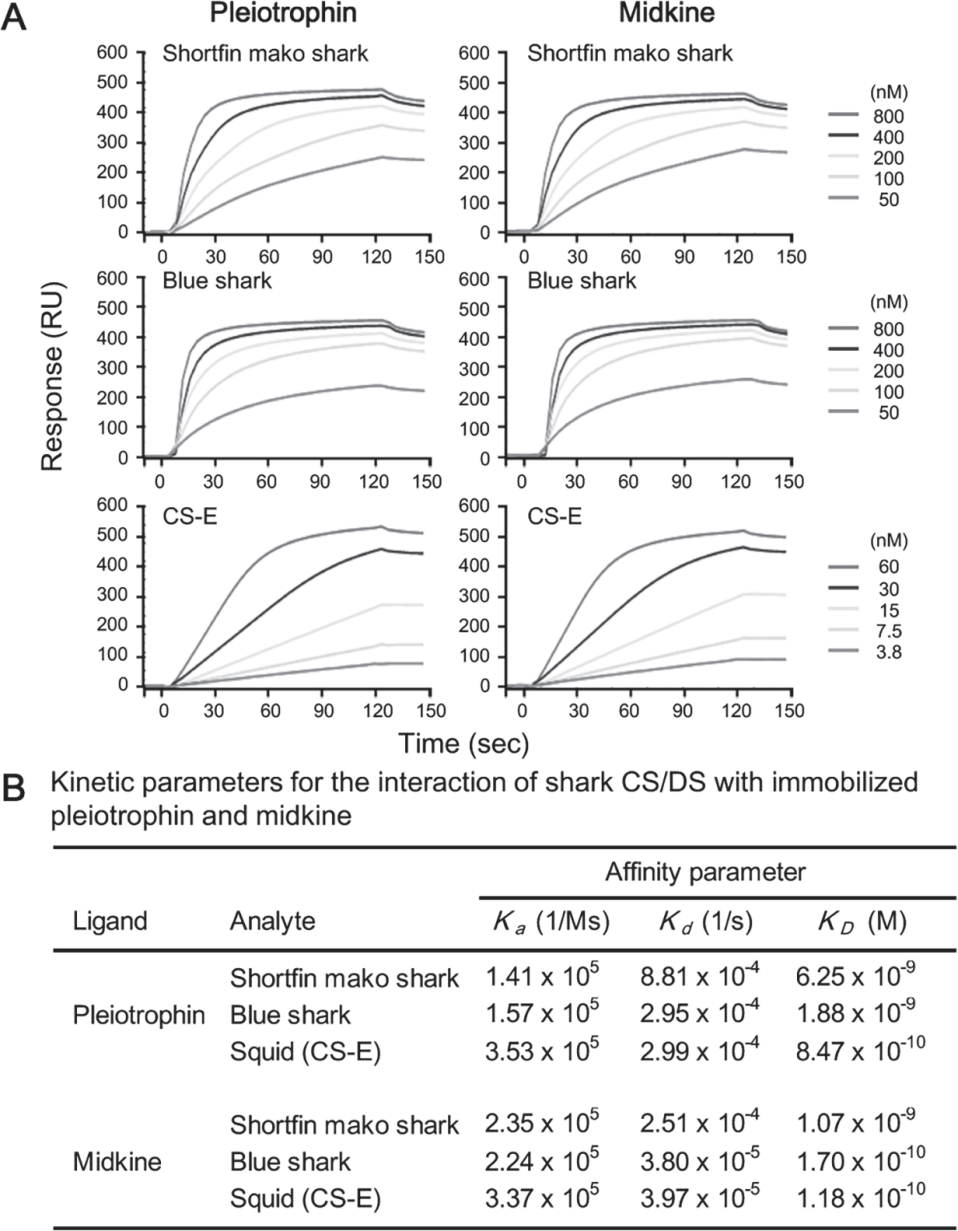
Binding of CS/DS from shortfin mako shark (Fr. 5) and blue shark (Fr. 3) to immobilize to growth factors. Various concentrations of shark CS/DS and squid CS-E (Seikagaku Corp., Tokyo, Japan) were injected onto the surface of a pleiotrophin- or midkine-immobilized sensor tip. Sensorgrams obtained with various concentrations of each shark CS/DS were evaluated using BIAevaluation 3.0 software. RU, resonance units.

Since shark CS/DS can stimulate the neurite outgrowth of embryonic mouse hippocampal neurons, we subsequently used a BIAcore system to analyze CS/DS interaction with selected growth factors to confirm the mechanism of neurite outgrowth promotion by shark CS/DS (Fig. 4A). Various concentrations of shark CS/DS or squid CS-E were submitted as an analyte onto surface of a sensor chip coated with pleiotrophin or midkine to determine the association and dissociation rate constants (*k*_*a*_ and *k*_*d*_) as well as the dissociation equilibrium constants (*K*_*d*_) (Fig. 4B). Squid CS-E displayed lowest *K*_*d*_ values but shark CS/DS showed substantial binding to growth factors. These results were consistent with the higher activity for neurite outgrowth observed for CS-E.

### Structural analysis of CS/DS derived from shortfin mako shark and blue shark

**Fig 5:**
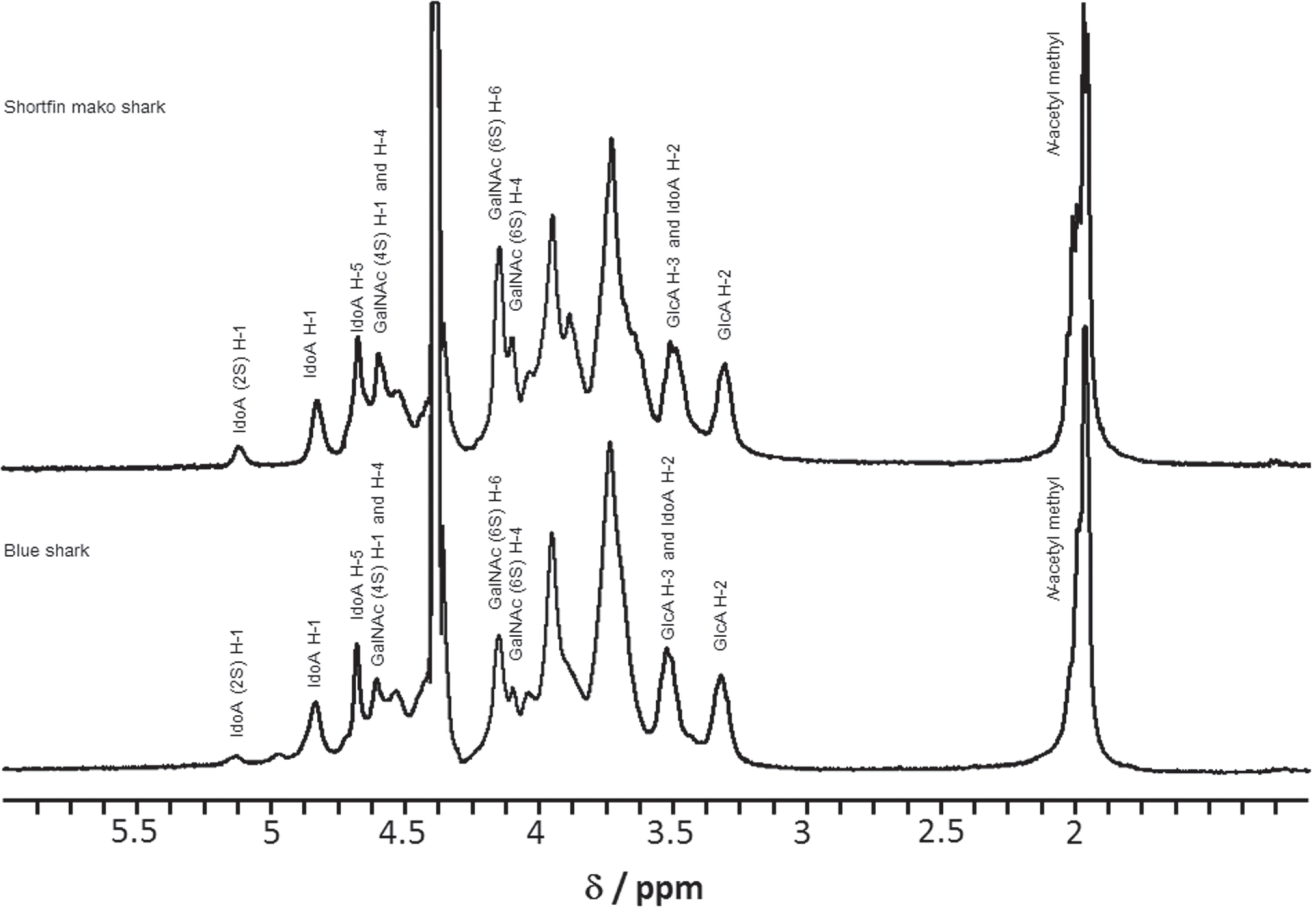
One-dimensional ^1^H-NMR spectra of CS/DS from shortfin mako shark (Fr. 5) and blue shark (Fr. 3).

One dimensional (^1^H) NMR spectroscopy is well known as one of powerful tools for determination of monosaccharide composition in polysaccharides [33]. ^1^H NMR spectroscopy was used to determine the percentage of GlcA and IdoA residues in CS/DS from Fr. 5 (shortfin mako shark) and Fr. 3 (blue shark) (Fig. 5). The anomeric H-1 (4.83 ppm), H-5 (4.63 ppm) and H-2 (3.52 ppm) signals of IdoA observed were similar to the signals seen in commercial DS from porcine skin or porcine mucosa [34]. The ratio of GlcA to IdoA in CS/DS from shark fin was different from porcine tissues. The two predominant peaks for the H-1 of IdoA (4.87 ppm) and the H-2 of GlcA (3.33 ppm) were used to determine the ratio of GlcA to IdoA. The composition of IdoA and GlcA in CS/DS was 41.2% and 58.8% (shortfin mako shark), 36.1% and 63.9% (blue shark), respectively.

**Fig 6:**
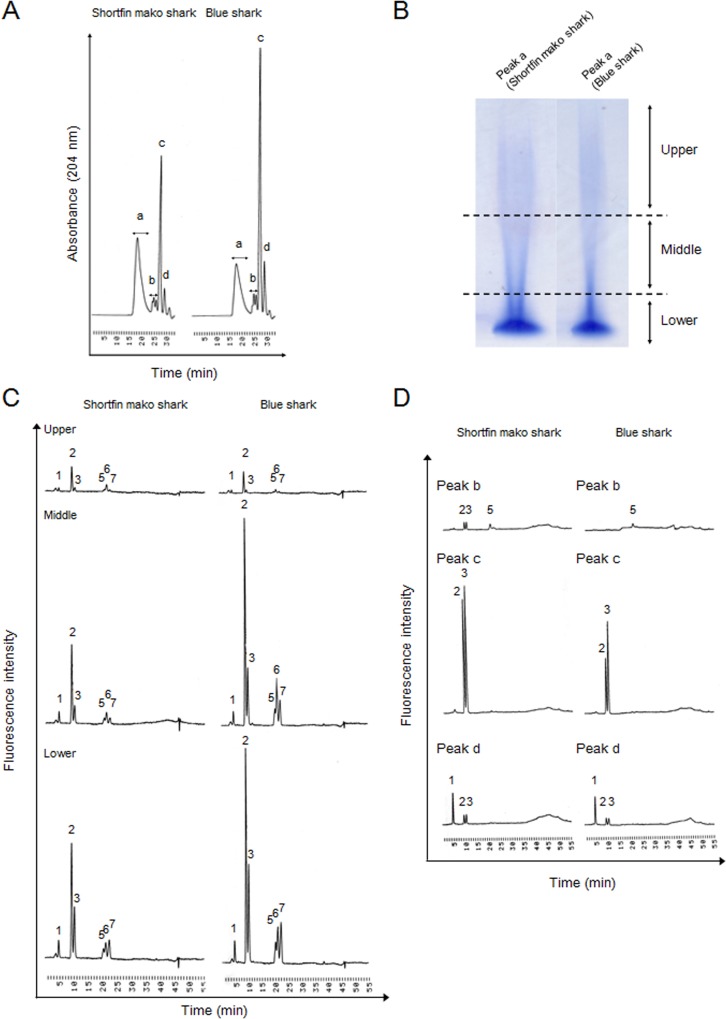
Determination of unsaturated disaccharide components in IdoA-rich fractions of different molecular weights. (A) Separation of IdoA-rich domain and partially degraded samples by ChaseACII. One mg of samples were subjected partial digestion with 1 unit of ChaseACII at 37°C for 1 h and then resulting degraded samples were fractionated by an HPSEC systems with an Asahipak 510HQ column (7.6 mm, i.d. × 300 mm) as described previously [27]. The fraction consisting of oligosaccharides contained an IdoA-rich domain as the GlcA-rich domains were degraded by ChaseACII. (B) Separation of IdoA-rich domain at the different molecular weight. Samples (0.1 mg) after digestion with ChaseACII were subjected by gradient SDS-PAGE (PAGEL NPG-1020L, 10–20%). The electrophoresis was performed as described previously [27]. (C) Determination of unsaturated disaccharides in IdoA-rich domain at the different molecular weight. (D) Determination of unsaturated disaccharides in fraction b, c and d in Fig. 6A. Peaks: 1, ΔDi-0S; 2, ΔDi-4S; 3, ΔDi-6S; 4, ΔDiUA-2S; 5, ΔDi-diS_E_; 6, ΔDi-diS_B_; 7, ΔDi-diS_D_.

**Fig 7:**
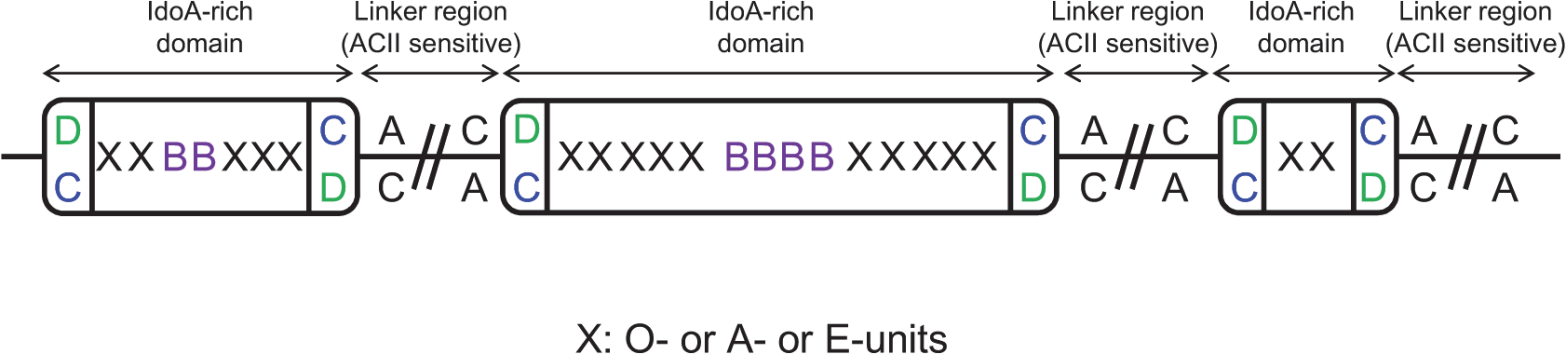
Proposed structures of shark CS/DS chains.

It has been reported that IdoA-rich domain exists in DS from mammalian tissues such as porcine skin decorin [24]. However, 4S disaccharide content of decorin DS is quite high (88%). Since shark CS/DS consisted of substantial quantities of other disaccharides, including ΔDi-6S, ΔDi-diS_E_, ΔDi-diS_B_ and ΔDi-diS_D_, shark CS/DS was partially depolymerized using ChaseACII to analyze oligosaccharide sequences. Oligosaccharide products rich in IdoA and the depolymerized sample was subsequently subjected to high-performance size exclusion liquid chromatography (**HPSEC**) chromatography (Fig. 6A). The fractions containing resistant oligosaccharides, enriched in IdoA (peak a), were collected and subjected to analysis by gradient (10–20%) polyacrylamide gel electrophoresis (PAGE) (Fig. 6B). The result of this analysis showed various lengths of IdoA-rich domains in shark CS/DS. The gradient gels were cut (as shown in the figure), crushed, and suspend in 2.5 M NaCl to isolate the different sized oligosaccharides. The pellets obtained after ethanol precipitation of these extracted oligosaccharides were desalted and dried. Disaccharide analysis of the different sized oligosaccharides was then performed after digestion with ChaseABC (Fig. 6C). Interestingly, the contents of ΔDi-diS_B_ (B-type units) in these IdoA-rich domains increased in a length dependent manner, but ΔDi-4S (A-type units) or ΔDi-diS_E_ (E-type units) were found uniformly throughout these IdoA-rich domains (Table 1). In contrast, the ΔDi-6S (C-type units) and ΔDi-diS_D_ (D-type units) contents of each fraction decreased in a molecular weight-dependent manner. These results suggested that C- or D-type units exist outside of IdoA-rich domains are present in small amounts in the IdoA-rich domains. Finally, the disaccharide analysis of degraded samples obtained in Fig. 6A was performed (Fig. 6D). The prominent disaccharide units of the most degraded samples were ΔDi-4S, ΔDi-6S and the minor disaccharide units were ΔDi-0S, ΔDi-diS_E_. The ΔDi-diS_B_ disaccharide was not observed. These results strongly indicate that linker region among IdoA-rich domain was composed of A- or C-type units (GlcA-rich domain). Based on the structural analysis using ChaseACII, it is possible to estimate on the disaccharide sequence in shark CS/DS (Fig. 7).

**Table 1:** Unsaturated disaccharide compositions of oligosaccharides containing IdoA-rich domain at the different molecular weight.

		Unsaturated disaccharide (%)						
		ΔDi-0S	ΔDi-4S	ΔDi-6S	ΔDi-diS_E_	ΔDi-diS_B_	ΔDi-diS_D_	ΔDi-TriS
Shortfin mako shark Fr. a	Upper	3.53	47.1	3.98	2.59	38.2	4.63	N.D.
	Middle	4.69	54.4	8.53	3.61	23.2	5.54	N.D.
	Lower	4.74	47.9	16.3	3.55	17.6	10.0	N.D.
Blue shark Fr. a	Upper	4.64	53.0	5.22	3.42	28.6	5.08	N.D.
	Middle	1.88	45.7	9.02	2.42	34.3	6.70	N.D.
	Lower	2.60	38.1	28.8	3.53	17.6	9.37	N.D.

N.D.: Not detected

## Discussion

We collected fins from 11 kinds of *Elasmobranchii* to better understand the biological distribution of GAGs and explore the sources of polysaccharides containing unique structures (Fig. 1). Commercially available CS is usually prepared from shortfin mako shark and blue shark cartilage. In fact, total GAG amount in blue shark cartilage (raw fin without skin) is the highest among 11 different *Elasmobranchii* examined (Fig. 1B). The disaccharide composition of the CS and the GAG recovery from red stingray fin were comparable to that of blue shark cartilage. These results suggest that red stingray fin might represent an alternative source of CS. CS proteoglycan, especially aggrecan is known to be present in the connective tissues together with HA, collagen, and other proteins and these biopolymers function to hydrate the tissue giving it gel-like and elastic properties. Therefore, we examined deep-sea shark cartilage, including birdbeak dogfish, kitefin shark, smalltooth sand tiger, goblin shark and spotless smooth-hound to identify the CS containing unique structures. However, the disaccharide composition of CS, from these species, was a typical form of CS-C or CS-D. Interestingly, degree of sulfation in CS was very low in frilled shark.

We also found that substantial amounts of CS/DS were contained in the dried fin (without skin and cartilage) of *Isurus oxyrinchus* and *Prionace glauca*. It has been reported that highly sulfated DS have been found abundantly in lower marine organisms and that these exhibit NOP activity and anticoagulant activity [13–17]. Shark CS/DS also showed NOP activity toward mouse hippocampal neurons, despite their lower degree of sulfation than other previously reported DS samples (Figs. 1B and 3). It seems that the ratio of di-sulfated disaccharide in DS rather than the sulfation pattern is important for its physiological function, as NOP activity was not observed for porcine skin DS, which has a mono-sulfated disaccharide (IdoA-GalNAc (4S)) as its predominant disaccharide unit (89%) [17]. In fact, the ratios of di-sulfated disaccharide in DS from lower marine organisms are higher than that of any other CS or porcine-derived DS. For example, IdoA-GalNAc (4S, 6S) (iE-type unit) is a major disaccharide found in DS from hagfish notochord (68%) and embryonic sea urchin (74%), while a significant amount of IdoA-GalNAc (2S, 6S) (iD-type unit) is found in Ascidian *A*. *nigra* DS (>90%) [14–16]. Interestingly, a high amount of IdoA-GalNAc (2S, 4S) (B-type unit) is also found primarily in DS from Ascidian *S*. *plicat* (66%) [13]. We suggest that oligosaccharides, consisting of a cluster of di-sulfated disaccharides, especially B-type units present in shark CS/DS, can interact with growth factors like midkine and pleiotrophin, which are important proteins for neurite outgrowth. Thus, it is important to analyze the oligosaccharide sequence of shark CD/DS containing substantial amount of di-sulfated disaccharides.

Sequence analysis of shark CS/DS was examined by partial digestion of ChaseACII (Fig. 6). Based on the structural analysis using ChaseACII and an understanding of the CS/DS biosynthetic pathway, we found that the IdoA and B-type units as well as A- and C-type units were present in clusters in shark CS/DS. Furthermore, it was discovered that the contents of B-type units in these IdoA-rich domains increased in a length dependent manner, while C- and D-type units were located particularly in the immediate vicinity of the IdoA-rich domain (Fig. 7).

It appears that the acceptor substrate specificity of the C5-epimerase and sulfotransferase enzymes is important determinants in the sequence of shark CS/DS. At first, the synthesis of CS and DS begins with polysaccharide chain extension through the action of β-*N*-acetylgalactosaminyltransferase-β-glucuronosyl transferases (Chys1, Chys2, and Chys3-like) [2,4]. The glucuronic acid residue in DS is C5-epimerized to iduronic acid through the action of DS epimerase. The 4-position of *N*-acetylgalactosamine (4S) of chondroitin is sulfated by chondroitin 4-*O*-sulfotransferases (C4ST-1 or C4ST-2) and same position of dermatan is sulfated by dermatan 4-*O*-sulfotransferase 1 (D4ST-1). The 4S sulfated product is subsequently sulfated at the 6-position by *N*-acetylgalactosamine 4-sulfate 6-*O*-sulfotransferase (GalNAc4S6ST) producing a disulfated product (E-type unit). In CS, the 6-position of *N*-acetylgalactosamine is sulfated by chondroitin 6-*O*-sulfotransferase 1 (C6ST-1) [2,35]. The presence of iC- and iD-type units in adult sea urchin and ascidian *A*. *nigra* have also been reported [14,16]. The GlcA residues of CS and the IdoA residues of DS can also be 2-sulfated by uronosyl 2-*O*-sulfotransferase (UA2OST).

In shark CS/DS, unsaturated disaccharides such as ΔDi-0S, ΔDi-4S and ΔDi-diS_E_ were detected in both the IdoA-rich, ChaseACII resistant region and also in the ChaseACII sensitive region (GlcA-rich domain) (Fig. 6 and Table 1). The ΔDi-6S was primarily detected ChaseACII sensitive domain, and the ratio of ΔDi-6S and ΔDi-diS_D_, in the IdoA rich domain, were inversely related to the molecular weight of the ChaseACII resistant oligosaccharides. In contrast, the distribution and content of B-units in the IdoA-rich domain was length dependent. These results are consistent with data suggesting that IdoA-GalNAc chain is poor acceptor substrate for C6STs [35] and DS is a better acceptor for UA2OST than CS-C [36]. The sequence, especially IdoA-rich domain of shark CS/DS, appears to be determined by DS epimerase activity and the acceptor substrate specificity of sulfotransferases. We have previously reported that composition of unsaturated disaccharide units in fractionated whale cartilage CS-A (7∼29 kDa) or in bovine tracheal cartilage CS-A (6∼33 kDa) obtained by gel filtration chromatography, was almost same in different molecular weight samples [27]. Furthermore, the composition of disaccharide units in fractionated shark cartilage CS-C (13∼47 kDa) was also almost same (data not shown). These results also support the idea that DS epimerase and acceptor substrate specificity of sulfotransferases decide the disaccharide sequence in CS/DS biosynthesis pathway. CS/DS have been identified in mammalian tissues and cells such as horse aorta [18], embryonic pig brain [19], mouse brain [20], mouse skin [21] and HEK293 cells [22]. Further analysis is required to understand the disaccharide unit sequence of mammalian CS/DS.

In summary, comprehensive analysis of GAGs among 11 kinds of *Elasmobranches* was carried out and found that characteristic disaccharide units of most CS were C- and D-type units. We identified and characterized CS/DS from dried shark fin (without skin and cartilage) in shortfin mako shark and blue shark. These CS/DS exhibited the NOP activity toward E16 embryonic mouse hippocampal neurons. Furthermore, we found that the IdoA and B-type units as well as C- and D-type units were found in clusters in shark CS/DS. In addition, it was found that the contents of B-type units in these IdoA-rich domain increased in a length dependent manner, while C- and D-type units were located particularly in the immediate vicinity of the IdoA-rich domain.

## Supporting Information

S1 Fig Electropherogram of crude GAGs from various kinds of *Elasmobranchii (shown in the upper portion of the figure)*. Lanes from left to right: A, *Scyliorhinus torazame*; B, *Chimaera phantasma*; C, *Deania calcea*; D, *Dalatias licha*; E, *Odontaspis ferox*; F, *Chlamydoselachus anguineus*; G, *Mitsukurina owatoni*; H, *Mustelus griseus*; I, *Dasyatis akajei*. GAGs (approx. 5 μg of each sample) were analyzed by electrophoresis on cellulose acetate membrane using 1 M pyridine-acetic acid buffer (pH3.5) at 0.5 mA/cm for 20 min. GAGs were stained by 0.5% alcian blue in 0.3% acetic acid.
(TIF)
