# Supplementary figures and images for: Composition of Glycosaminoglycans in Elasmobranchs including Several Deep-Sea Sharks: Identification of Chondroitin/Dermatan Sulfate from the Dried Fins of Isurus oxyrinchus and Prionace glauca
Source: PLoS One. 2015 Mar 24;10(3):e0120860. doi: 10.1371/journal.pone.0120860 (PMC4372294; doi:10.1371/journal.pone.0120860)

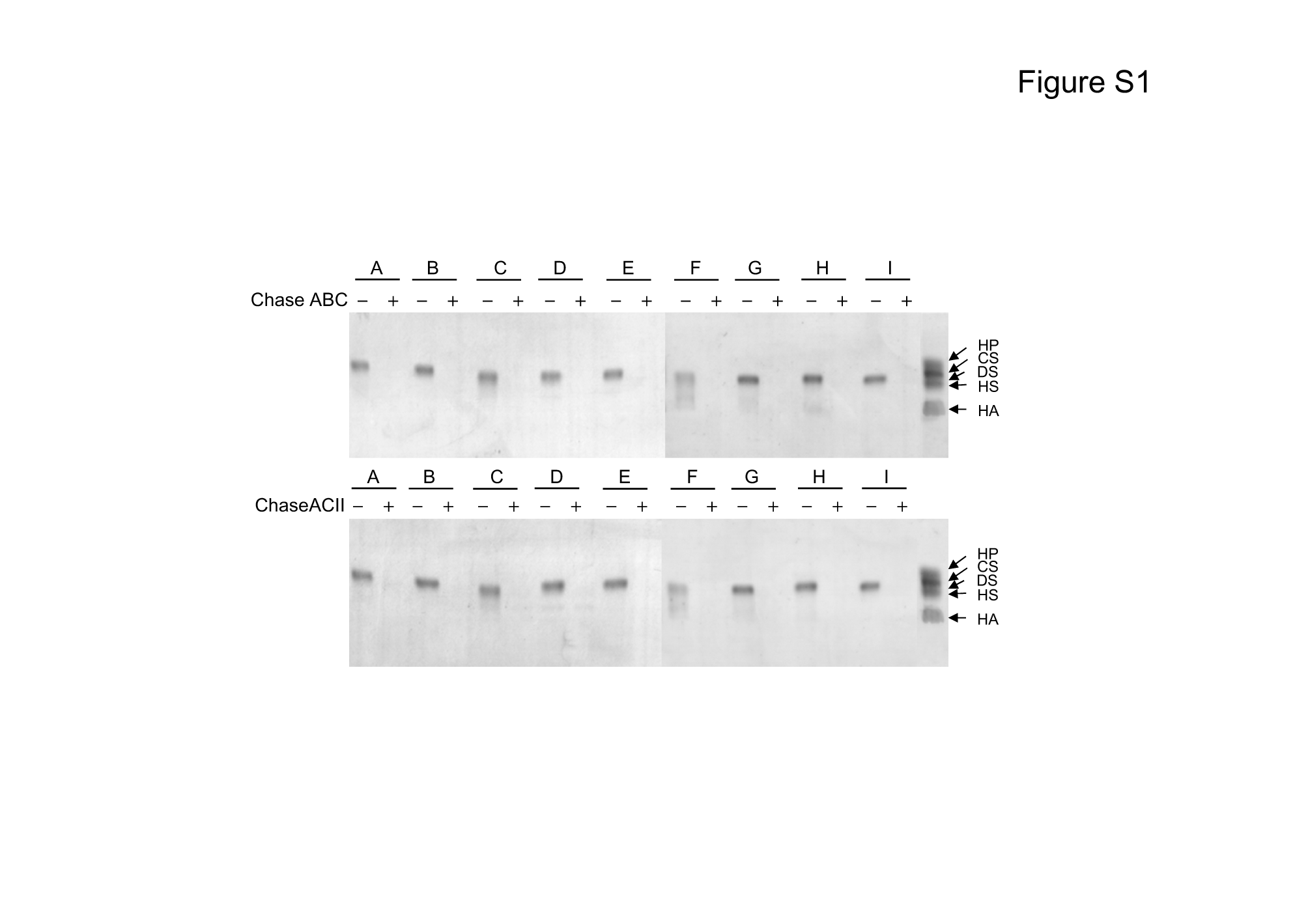

Supplement: S1 Fig — Lanes from left to right: A, Scyliorhinus torazame; B, Chimaera phantasma; C, Deania calcea; D, Dalatias licha; E, Odontaspis ferox; F, Chlamydoselachus anguineus; G, Mitsukurina owatoni; H, Mustelus griseus; I, Dasyatis akajei. GAGs (approx. 5 μg of each sample) were analyzed by electrophoresis on cellulose acetate membrane using 1 M pyridine-acetic acid buffer (pH3.5) at 0.5 mA/cm for 20 min. GAGs were stained by 0.5% alcian blue in 0.3% acetic acid. (TIF) [file pone.0120860.s001.tif]
